# Experiences of Food Insecurity Among Pregnant Adolescents and Adolescent Mothers in Ghana: A Photovoice Method

**DOI:** 10.3389/ijph.2024.1607043

**Published:** 2024-05-14

**Authors:** Isabelle Posey, Christiana Nsiah-Asamoah, Nicholas Fergusson Russell, Esther Darkwa Entwi, Harriet Okronipa

**Affiliations:** ^1^ Department of Nutritional Sciences, Oklahoma State University, Stillwater, United States; ^2^ Department of Clinical Nutrition and Dietetics, University of Cape Coast, Cape Coast, Ghana

**Keywords:** food insecurity, nutrition, adolescent girls, pregnancy, adolescent motherhood, Photovoice, Ghana

## Abstract

**Objectives:** Food insecurity (FI) remains a major public health problem globally. However, there is limited information about adolescents’ experiences. The current study explored FI experiences of pregnant adolescents and adolescent mothers in Cape Coast, Ghana using a Photovoice method.

**Methods:** This study recruited 34 pregnant adolescents and adolescent mothers from communities in Cape Coast, Ghana. Each participated in a training session then was provided prompts to take photos that portrayed food access barriers, facilitators and coping strategies. In a debrief session, each participant selected two pictures they took and explained the image, followed by a group discussion of the selected photos. Debrief sessions were audio recorded and transcribed verbatim to develop themes using a theory-driven approach.

**Results:** Most participants reported several or many experiences with FI (64.7%) in the previous month. Participants discussed money, unwanted pregnancy, and unstable work as barriers to obtaining food and working, selling goods, and family support as facilitators to obtaining food. Coping strategies mentioned include providing services to others, borrowing food and goods, meal stretching, pawning personal items and trading.

**Conclusion:** The FI experience of this population is complex; interventions, including trade training or school retention, should be multifaceted.

## Introduction

Food insecurity is a persistent global health concern [1]. In 2021, the Food and Agricultural Organization (FAO) estimated that around 2.3 billion people were moderately or severely food insecure [2]. Countries with higher poverty rates experience the burden of food insecurity disproportionately. In Ghana, 49% of the population faced food insecurity in 2022 [3]. Notably, nearly three-quarters of Ghanaian adolescents are food insecure, with female adolescents at a slightly higher risk than males [4]. Adolescence is a critical period for growth and development and adequate nutrition during this period is essential. Within the demographic group of adolescents, a particularly susceptible sub-group comprises pregnant adolescents and teenage mothers, often referred to as a marginalized and forgotten group facing severe challenges. They face significant hardships due to financial struggles, impacting their ability to access food.

Food insecurity during pregnancy and early motherhood can have deleterious impacts on the health and nutrition of both mother and child [5–7]. Additionally, children born to adolescent mothers face an increased risk of nutritional deficiencies, perpetuating into an intergenerational cycle of malnutrition, as highlighted in a recent systematic review and meta-analysis [8]. In Ghana, adolescent pregnancies are prevalent. According to the 2022 Ghana Demographic and Health Survey, 15% of Ghanaian adolescents aged 15–19 years have started childbearing with about an 11% live birth rate [9]. While food insecurity remains an important problem, there is limited research exploring the food insecurity experiences among pregnant adolescents and adolescent mothers in Ghana. Additionally, the barriers, facilitators, and coping strategies related to food access among this population is poorly understood.

Photovoice, a visual participatory research method, has emerged as a valuable tool for exploring and addressing important public health issues. It empowers participants to share their reality and tell their story rather than have it told [10]. Photovoice has unique advantages as a qualitative research method, especially in global research as it is easy for participants to learn and engage in, and the resulting photos are worth a thousand words that can cross culture and language divides [11]. A study conducted in three African cities utilized Photovoice to identify food environment factors that impacted dietary behaviors in low-income populations [12]. Photovoice was used in another study in Ghana to examine individual-level drivers of diet behaviors in women 13–49 years of age. This study found that facilitators and barriers included income, nutrition knowledge, cooking skills, and time constraints [13]. Other studies in Ghana have used this tool to explore the lived experiences of adolescent mothers [14].

The current study aims to address this gap in the literature by using a photovoice method to understand the barriers, facilitators, and coping strategies related to food access among a population of pregnant adolescents and adolescent mothers in Ghana. By engaging participants in the research process and allowing them to share their experiences through photography and group discussions, we hope to gain some insights into the unique experiences of food insecurity faced by this underserved population. This is important to inform the development of contextually appropriate interventions to improve food insecurity among this group.

## Methods

### Study Setting and Design and Population

This cross-sectional, Photovoice study was conducted in Cape Coast, Ghana between May and July 2023 as part of the Healthy Adolescents in Ghana Study (HANIG II). Participants were recruited from seven peri-urban communities purposely selected from 33 communities in the study area. Communities were selected to reflect diversity in socioeconomic and demographic characteristics. All seven communities had a relatively large adolescent population and consisted of low-income households who were mainly fisherfolks or farmers. Individuals were eligible to participate if they were between ages 12 and 19 years and were pregnant or had a child under age 5 years. Eligible participants were taken through an informed consent process where the purpose and voluntary nature of the study was clearly explained to them in simple language tailored to their age and comprehension level. To ensure comprehension of the information, the entire study was verbally reviewed for the participants in the local language (using the consent form as a guide). Additionally, time was allowed for questions, and questions were also asked of the participants during the consent process to gauge their understanding. Participants who expressed interest and gave their consent (or parental consent) were then enrolled and invited to participate in every part of the study. This study was approved by the Institutional Review Boards of Oklahoma State University, United States and the University of Cape Coast, Ghana.

### Data Collection Procedures

After enrollment, data on socio-demographic characteristics and food insecurity were collected in the participant’s home by means of interviewer-administered questionnaires. Food insecurity was assessed using the Child Food Insecurity Experience Scale, a 10-item scale that asks about a child’s food insecurity experiences in the month preceding the interview. Response options for each item on the questionnaire range from 0 to 2 (0, never; 1, one or two times; 2, many times). All study questionnaires were administered by trained field workers, recruited from the study communities and who spoke the local languages of the study area -Twi and Fante.

Information on the barriers, facilitators and coping strategies related to food access were collected using the photovoice method, a participatory research method that empowers participants to visually express their experiences [10] using a digital camera. This data collection method is easy for participants to learn and engage in, and the resulting photos can cross culture and language divides [11]. All sessions were held at a central place within each community, conducted in the local language by trained field workers, and consisted of three main activities—a training session, a photo-taking activity, and a discussion session.

#### Photovoice Training Session

At the first training session, participants were taken through 1) the photovoice concept 2) the use of a camera to take different types of pictures in response to specific questions and 3) the ethics involved in taking photos, including the need to obtain consent before taking any photographs. To ensure they understood, participants were given several example questions/prompts (unrelated to the study objectives) and asked to practice taking photos based on these prompts to learn how to use the camera and practice using pictures to communicate their thoughts.

At the end of the training session, each participant was assigned a camera to take five photographs over a 2-day period that best represented 1) something that makes it difficult for them to provide food for themselves and their household 2) something that makes it easy to provide food for themselves and their households and 3) coping strategies they use when food or money is limited.

#### Photovoice Debrief Session

All cameras were collected the day prior to the debrief session and all photos were downloaded and printed (two copies of each). The debrief session consisted of an individual interview session, followed by a group discussion session.

##### Individual Interview

Each participant’s printed photos were shown to them, and they were instructed to select two they wanted to discuss. For each selected picture, the participant was requested to provide a short caption explaining why they took that picture.

##### Group Session

The group session was led by two fieldworkers, a facilitator, who led all discussions, and a note-taker. All selected photos were posted on a flipchart labeled with the appropriate prompt, so they were visible to all. Addressing one prompt at a time, each photo was discussed using the SHOWeD method. Specifically, participants were asked: a) What do you SEE in the photo? b) What is HAPPENING? c) How does this relate to OUR lives? d) WHY does this situation or concern exist? and e) What can be DONE to fix these problems?

One photo was discussed entirely before moving to the next.

### Data Analysis

Each Photovoice session was audio recorded and transcribed verbatim, excluding any personal identifiers of participants. Three members of the research team coded and analyzed the transcripts using a theory-driven approach. Possible themes were first established based on relevant literature on the barriers, facilitators, and strategies of food insecurity experience. Coding units consisted of overarching themes (barriers, facilitators, and strategies), subthemes, and quotations of entire narratives that depict selected pictures taken by the participants. Common subthemes in each overarching theme were identified by finding the frequency at which each subtheme was quoted by unique participants.

## Results

**FIGURE 1:**
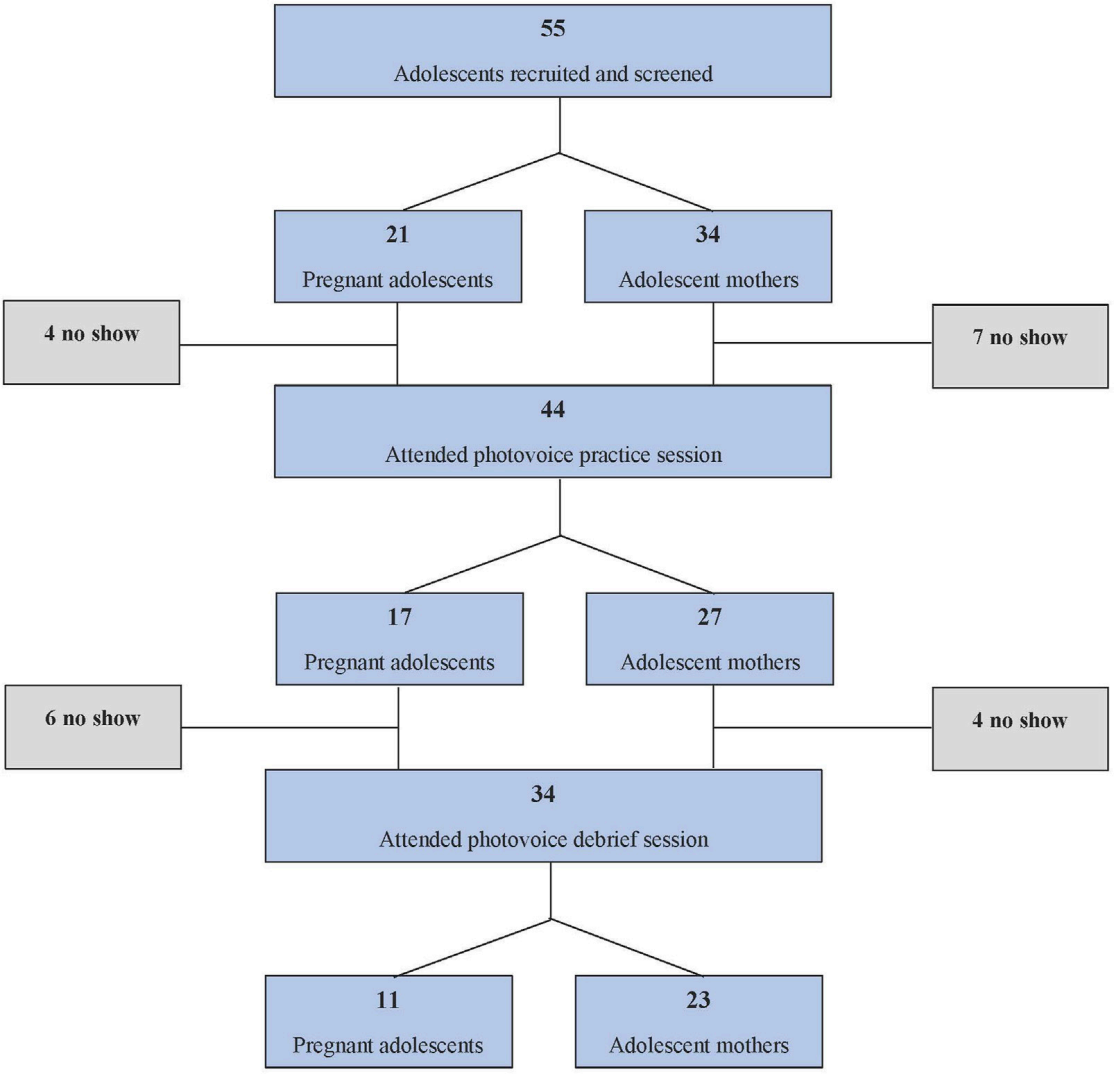
Study flow chart (Healthy Adolescents Nutrition in Ghana study, Cape Coast, Ghana, 2023).

A total of 34 adolescent mothers (*n* = 23) and pregnant adolescents (*n* = 11) participated in all photovoice sessions and were included in the analysis (Figure 1). On average, participants were 18 ± 1.1 years old with 26.5% enrolled in school. Most (65%) reported several or many experiences with food insecurity in the last month. About three-quarters of them were unemployed and their primary income source was from their parents (29%) or their child’s father (21%) (Table 1).

**TABLE 1:** Socio-demographic characteristics of the photovoice participants (Healthy Adolescents Nutrition in Ghana study, Cape Coast, Ghana, 2023).

	Mothers (*n* 23)		Pregnant (*n* 11)		Overall (*n* 34)	
	n	%	n	%	n	%
No FI^a^ experience	1	4	0	0	1	3
Any FI experience	23	96	10	100	33	97
Few FI experiences	6	25	5	50	11	32
Several FI experiences	3	13	2	20	5	15
Many FI experiences	14	58	3	30	17	50
Enrolled in school	3	13	6	60	9	27
Married	1	4	0	0	1	3
Cohabitating	3	13	1	10	4	12
Income source						
Employed	5	21	1	10	6	18
Partner/child’s father	5	21	2	20	7	21
Parents	5	21	5	50	10	29
Relatives	2	8	2	20	4	12
Relatives of partner	5	21	0	0	5	15
Benevolent group	1	4	0	0	1	3
Other	1	4	0	0	1	3
Employment						
Not employed	16	67	9	90	25	76
Seamstress	1	4	0	0	1	3
Trader	1	4	0	0	1	3
Hairdresser	3	13	1	10	4	12
Other	3	13	0	0	5	15
Number of children						
0	0	0	10	100	10	29
1	19	79	0	0	19	56
2	5	21	0	0	5	15

^a^ Food Insecurity.

### Theme 1 Barriers

#### Money

**FIGURE 2:**
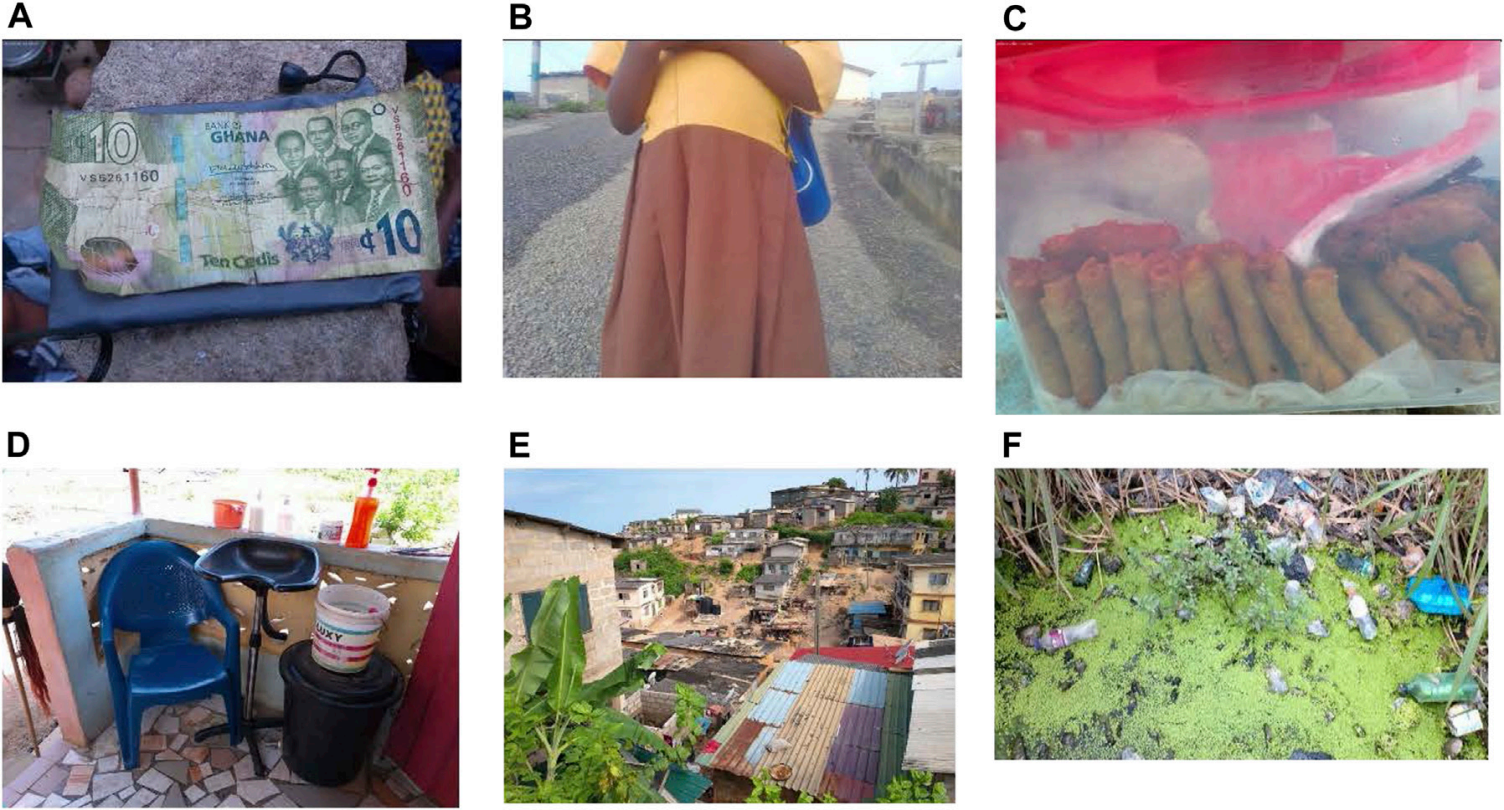
Pictures and captions depicting barriers to food access (Healthy Adolescents Nutrition in Ghana study, Cape Coast, Ghana, 2023): **(A)** “This 10 cedis note I have taken a picture of, represents the difficulty in finding money to buy food” (Pregnant adolescent, Community 1); **(B)** “Some teachers even made fun of my pregnancy when I was in school, and this really made me feel uncomfortable.” (Adolescent mother 5, Community 7); **(C)** “Absence of the product renders me unable to afford food.” (Adolescent mother, Community 4); **(D)** “If people don’t come to get their hair done, I won’t get money to buy food.” (Adolescent mother 1, Community 6); **(E)** “There is no place in the community where someone can decide to mount a shop to sell food.” (Pregnant adolescent 4, Community 3); **(F)** “Stagnant water with rubbish will prevent us from planting our crops and even interfere with their growth.” (Adolescent mother 1, Community 6) (Healthy Adolescents Nutrition in Ghana study, Cape Coast, Ghana, 2023).

Money was the most mentioned barrier affecting food access. This subtheme was brought up by 39% of the participants in focus group discussions. Throughout the discussion, participants expressed that without it, it is impossible to provide for themselves and their family. Participants in general held the belief that if they had more financial assets, they would have no issue obtaining food (Figure 2A). One participant said, *“It could be that food is available to buy but there is no money to buy it”* (Pregnant adolescent 2, Community 3).

#### Unwanted Pregnancies

The incidence of unwanted pregnancies was another common subtheme that 19% of participants mentioned as a barrier (Table 2). The participants discussed how they faced ridicule and stigma when they were pregnant (Figure 2B). This oftentimes resulted in the participants feeling unwelcome and uncomfortable in school, and sometimes leading to them dropping out. Two participants narrated their experience using Figure 2B:

*“Some teachers even made fun of my pregnancy when I was in school, and this really made me feel uncomfortable.”* (Adolescent mother 5, Community 7)

*“Getting pregnant in school made me quit school.”* (Adolescent mother 4, Community 7)

**TABLE 2:** Prominence of subthemes (Healthy Adolescents Nutrition in Ghana study, Cape Coast, Ghana, 2023).

Barriers	
Money	39%
Unwanted pregnancy	19%
Unstable jobs	13%
Improper building settings	10%
Footwear	7%
Lack of fertile land	7%
Lack of food purchasing aid	3%
Lack of support	3%
Unfavorable work environment	3%
Facilitators	
Finding work	41%
Selling goods	30%
Support from family	8%
Money	5%
Transactional sex and coerced relationships	5%
Clean water	3%
Borrowing	3%
Child marriages	3%
Cooking	3%
Strategies	
Providing services to others	25%
Borrowing	13%
Meal stretching	13%
Pawning personal items	13%
Trading/selling	13%
Obtaining food from items sold	13%
Opting for cheaper food	6%
Backyard garden	6%

Their pregnancies also impacted their ability to work, the support received from the community, and the allocation of resources they did have. Many participants expressed beliefs that their unwanted pregnancy made life in general harder for them. The following narrations below depict their experiences:

*“No one was willing to help me out when I was pregnant, so getting money for food was an issue.”* (Adolescent mother, Community 7)

*“She had money to run her personal business but when she gave birth, life has been quite difficult for her because she needs to take care of the child.”* (Adolescent mother 8, Community 7)

*“As a result of our pregnant states, our supporters might be deterred from helping us. For instance, if I want to go back to school, the person who has the capacity to support me might not do so because he or she might see me as useless now that I am pregnant”* (Pregnant adolescent 4, Community 3)

#### Unstable Jobs

Of total participants, 13% mentioned unstable jobs as a barrier to providing food (Table 2). Many of the participants mentioned utilizing some form of work or trade to provide for themselves and their families (Figures 2C, D). However, a common barrier among participants was the instability of their work and that of people in the community who support them. Despite efforts and skills, without the business they cannot provide for themselves. Participants narrated their experiences using Figures 2C, D.

*“Absence of the product renders me unable to afford food.”* (Adolescent mother, Community 4)

*“If people don’t come to get their hair done, I won’t get money to buy food.”* (Adolescent mother 1, Community 6).

#### Infrastructure

The subtheme of building infrastructure was mentioned by 10% of participants as a possible barrier to food attainment (Table 2). The participants mentioned the lack of community development and infrastructure planning as barriers to obtaining food (Figure 2E), as noted in the following quotes:

*“There is no place in the community where someone can decide to mount a shop to sell food.”* (Pregnant adolescent 4, Community 3)

*“The distance from one’s house to the food joint might be far, causing the pregnant teenager to be tired.”* (Pregnant adolescent 4, Community 3)

*“When I need to go out, I have to pass through tight corners to be able to go and that makes it difficult for me to go out and buy food.”* (Pregnant adolescent, Community 3)

#### Infertile Land

The lack of fertile land was a mentioned barrier of food obtainment by 7% of participants (Table 2). Participants expressed how the state of their land and water is a barrier to providing food for themselves and families (Figure 2F). Because of the environmental condition they could not take advantage of the land and grow food. The following quotation expands on this problem.

*“If not for this, we can do a plantation if we don’t have money for food.”* (Adolescent mother 1, Community 6)

### Theme 2 Facilitators

#### Finding Work

**FIGURE 3:**
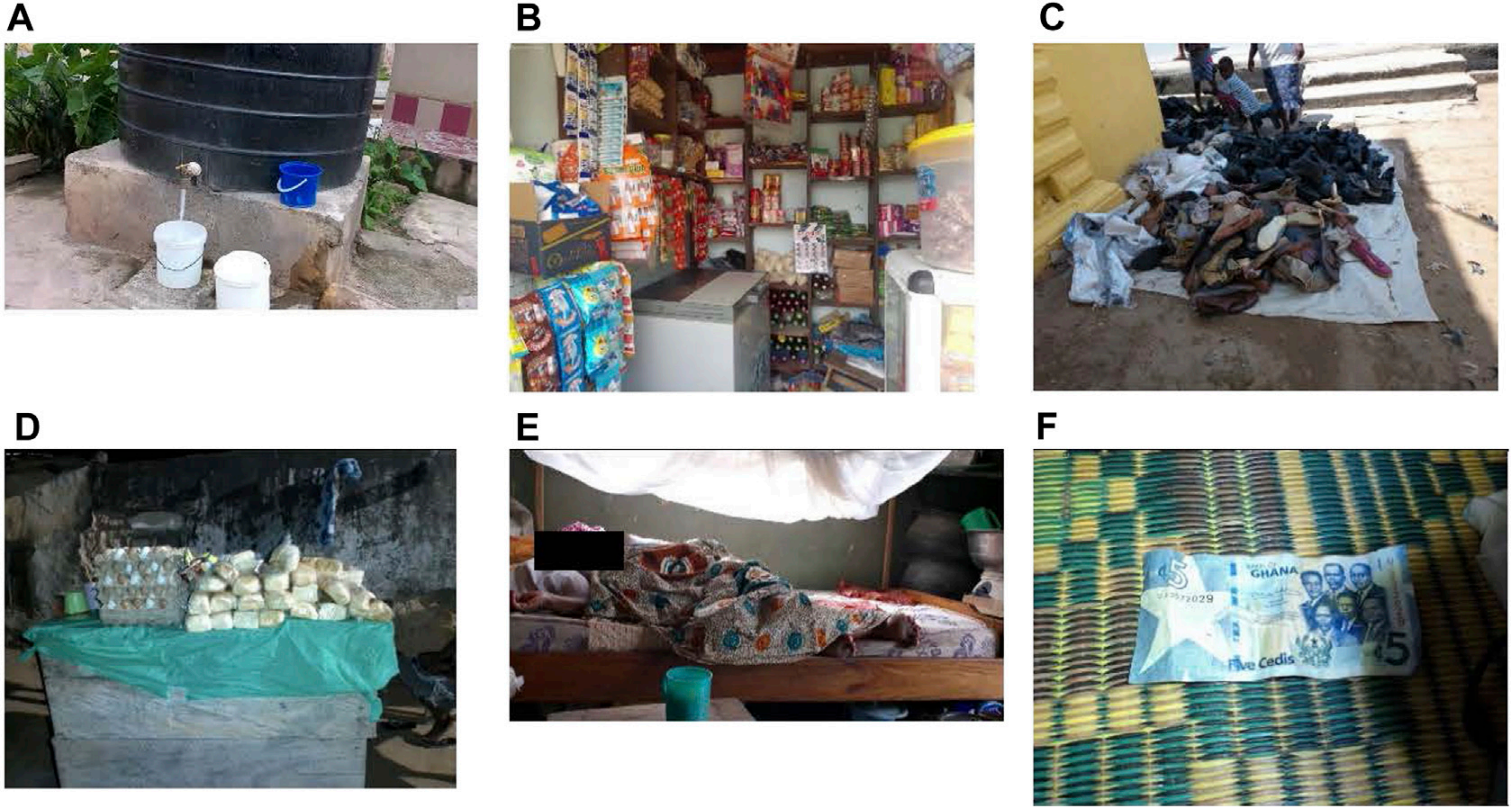
Pictures and captions depicting facilitators to food access (Healthy Adolescents Nutrition in Ghana study, Cape Coast, Ghana, 2023): **(A)** “If the general tap is closed, you can sell the stored water for money.” (Adolescent Mother 4, Community 2); **(B)** “I am a shop attendant and I get money to feed myself through this avenue.” (Adolescent mother, Community 7); **(C)** “I go to this place where I am given some footwear. This is what I sell for a living.” (Adolescent mother, Community 7); **(D)** “In times of difficulty, I help out my aunt who sells bread and eggs to make her sales when she is not around. This helps me both get food to eat and money for my upkeep.” (Adolescent mother, Community 7); **(E)** “If I stay with my grandmother, my dad gives me money for food and I also get food from her.” (Adolescent mother, Community 4); **(F)** When there is no food and there is money, it helps in purchasing food to curb hunger” (Pregnant adolescent 4, Community 1) (Healthy Adolescents Nutrition in Ghana study, Cape Coast, Ghana, 2023).

When the participants were asked about what helps them obtain food for themselves and their families the most mentioned subtheme was the ability to find work. Of the participants that talked about facilitators, 41% discussed how finding work is what helps them find food. There were many different types of work mentioned under this subtheme, such as washing clothes, braiding hair, or working in shops but the biggest facilitator for this population was finding some avenue of work (Figures 3A–D). Along the same lines, 30% of participants mentioned selling goods as a way to facilitate getting food. Some received support from their family that would give them items to sell. Using Figure 3C, one participant said:

*“This picture reminds me of my pregnancy. I used to sell mangoes when I was pregnant in order for me to get money for food.”* (Adolescent mother 8, Community 7).

#### Family Support

In discussion, 8% of participants mentioned the support received from their families as a way to help them get food. A few talked about receiving financial benefits from their family if the adolescent abided by specific living standards (Figure 3E). One participant said: *“Some fathers will send money to you provided you are not staying with your baby’s father.”* (Adolescent Mother 1, Community 4).

#### Money

Participants also discussed money as a facilitator, not just a barrier (Figure 3F). About 5% of participants discussed money as a facilitator to food acquisition. They talked about how having money enables them to feed themselves (Figure 3F).

#### Transactional Sex and Coerced Relationships

Finally, 5% of participants also discussed how transactional sex and coerced relationships were facilitators to providing food for themselves and their family. It was mentioned as a way to get money (Figure 3F) and support from men or that their parents push them into the position to alleviate the financial burden on the family. In response to Figure 3F, some participants said:

*“Also, some parents push their children towards men for money in order to reduce the burden on them.”* (Pregnant adolescent 4, Community 3)

*“Some men do not give for free. You would have to have sex with them so as to get the money.”* (Pregnant adolescent 4, Community 3)

### Theme 3 Strategies

#### Providing Services

**FIGURE 4:**
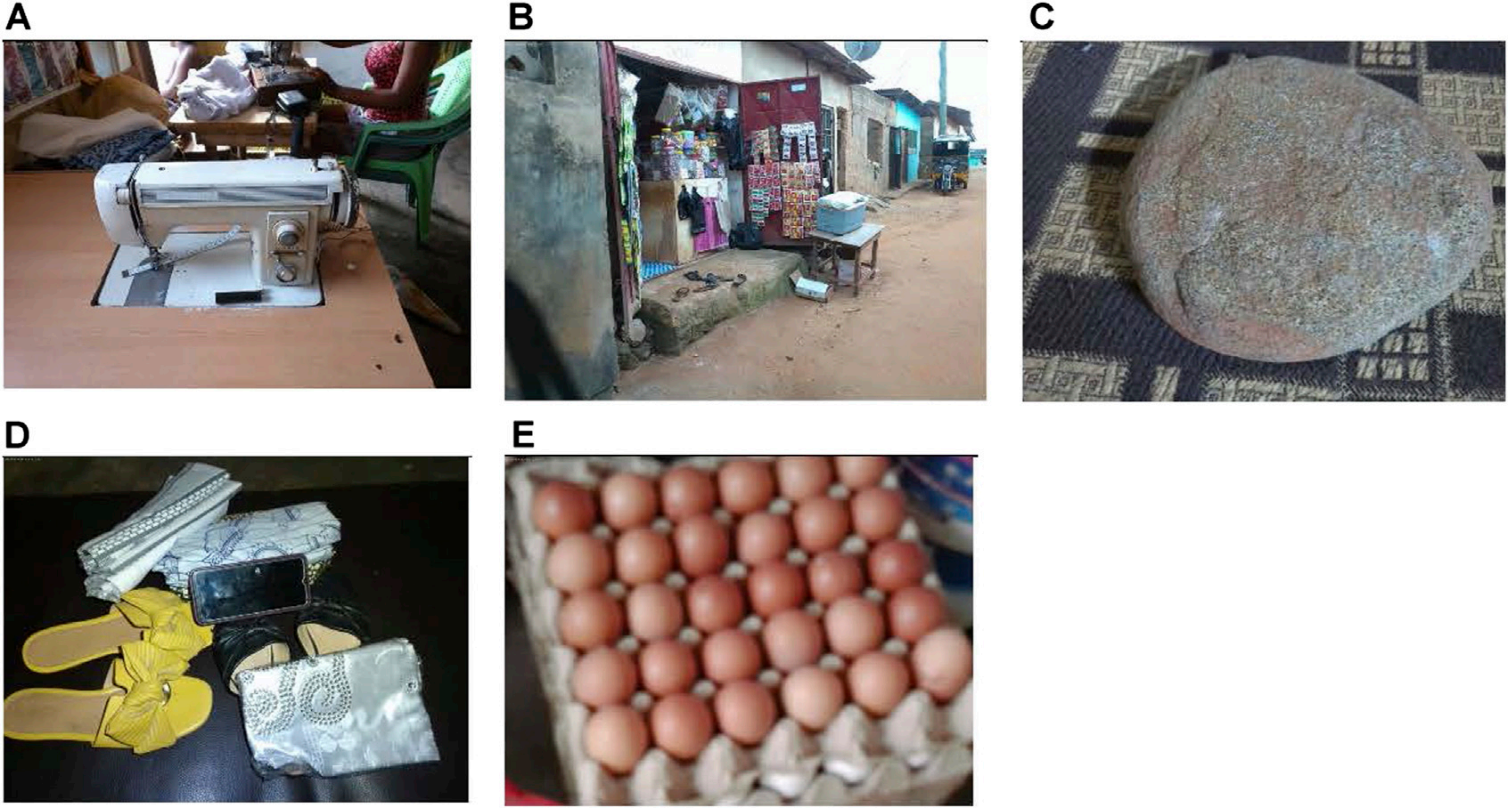
Pictures and captions depicting coping strategies (Healthy Adolescents Nutrition in Ghana study, Cape Coast, Ghana, 2023): **(A)** “Acquiring a handiwork of being a seamstress to be self-dependent” (Adolescent mother, Community 2; **(B)** “When I am hungry and have no food, I go and borrow gari from a woman who sells some. When I get enough money, I pay her back and the process continues.” (Adolescent mother, Community 6); **(C)** “This is how I get food to eat. When I want two of something and I can only get or afford one, I have to manage the one that I have like that. That is what this stone symbolizes (symbolizes having one of something).” (Pregnant adolescent, Community 3); **(D)** “We can sell the items and use the income for anything we need including feeding” (Pregnant adolescent 4, Community 3); **(E)** “If I have a store, I can pick stuff from the store to eat when I am hungry” (Adolescent mother, Community 6) (Healthy Adolescents Nutrition in Ghana study, Cape Coast, Ghana, 2023).

When participants were asked about strategies they used to obtain food, the most mentioned strategy was providing services to others which was mentioned by 25% of participants. They discussed how finding work can lead to independence. Many ways of providing services were mentioned, like being a seamstress (Figure 4A) or charging people’s phones for money, but the participants also mentioned that this can be a source of barriers. If they are missing the tools, machinery, or even experience needed to perform these services then they cannot make money this way. One participant said:

*“The machine is used to sew for money. If I don’t have the machine, I can’t work for money to buy food.”* (Adolescent mother 4, Community 2)

#### Borrowing Foods and Goods

The next strategy was borrowing, and this was discussed by 13% of participants. The participants mentioned borrowing food to eat and paying them back when they had the funds or borrowing goods from other people to sell for money (Figure 4B). One participant said:

*“It reminds me of when I had no money for food. I could get what I want if I had a shop or borrowed food stuff from someone’s store.”* (Adolescent mother 5, Community 6)

#### Meal Stretching

Meal stretching was mentioned by 13% of participants. They talked about how they learn to manage with less food than needed (Figure 4C). The strategies used in this case were buying more when the funds were there, eating less than needed, or buying cheaper proteins like eggs. The following quotes expand on this subtheme.

*“I purchase everything such as foodstuff in advance when I have a little money. This prevents me from running out of food like I normally would if I did not buy in advance.”* (Pregnant Adolescent, Community 3)

*“When what you have is not enough, you will have to manage what you have till you get more.”* (Pregnant adolescent 2, Community 3)

#### Pawning Personal Items

Pawning personal items was another strategy mentioned by 13% of the participants in discussion. Similar to borrowing, this strategy includes the adolescents selling their personal items for money to buy food (Figure 4D).

*“Sometimes, you might see something that you are interested in but you do not have enough money. You will have to sell these items in order to buy what you need”* (Pregnant adolescent 5, Community 3)

#### Selling Goods

Finally, participants (13%) mentioned trading/selling as a coping strategy. They sold goods to buy food and to take care of themselves (Figure 4E). Other strategies not mentioned as often include utilizing a backyard garden (6%) or buying cheaper food items (6%). The following quotes expand on the selling strategy:

*“When I sell, I get money to buy food and fend for my upkeep. Without selling, I cannot get money for my upkeep.”* (Adolescent mother, Community 4)

## Discussion

### Barriers

This study identified money, unwanted pregnancy, unstable jobs, building infrastructure, and lack of fertile land as barriers to food access. Money was the most discussed barrier, understandably as most participants were unemployed at the time of the study. Money is a theme seen often throughout literature as a force behind food insecurity. In similar studies, teenage mothers described the financial constraints that came with motherhood as the greatest barrier to meeting their and their child’s nutritional needs [14–17]. Similar findings have been reported in other studies in Eritrea [18] and the United States [19, 20].

Unstable jobs were also mentioned as a barrier to food access, which is not surprising considering that most adolescent mothers cannot secure high-paying job because they have not yet attained the educational qualification or skill [21, 22]. Inconsistent work may impact income, which may increase the risk for food insecurity. A study identified household heads that work varied hours, a marker of unstable work, to be more likely to be food insecure [23].

Unwanted pregnancy was also mentioned as a barrier in the present study. This finding is supported by a cross-sectional study in Iran where unplanned pregnancies in adult women were associated with two times higher odds of food insecurity than women with wanted pregnancies [24]. This is likely related to the fact that adolescents are unprepared for the unwanted pregnancies and do not have appropriate arrangements available. They will then have the struggle of finding supervision for their child to work and have more dependents to provide food for.

Lastly, participants mentioned the lack of fertile land as a barrier. This issue has been discussed in another Photovoice study in Ghana focusing on illegal mining activities popularly referred to as galamsey, or small scale gold mining [25]. In that study participants shared their experience with growing foods in galamsey-covered pits. For instance, mercury deposits in the land can seep into tubers of cassava and can make people sick when consumed. The mining also impacts bodies of water and aquaculture, effectively impacting all areas of food availability. This lack of fertile land impacts the pregnant adolescents and adolescent mothers by limiting the use of their land to provide for themselves. Many mentioned that if they had access to fertile land, they would grow their own food. The infrastructure and lack of planning of community development is another aspect of the environment that was identified as limiting food security in this population.

### Facilitators

Facilitators that were discussed in focus group sessions include the ability to find work, money, selling goods, family support, and transactional sex. The ability to find work as a facilitator to food access is in line with findings that unstable jobs are related to food insecurity. The ability to find work is directly tied to the opportunity to make money, another facilitator identified, and provide for a household. Related studies conducted among teenage pregnant girls and adolescent mothers revealed that some of them as a result of financial constraints engaged in menial jobs such as helping with sales in shops, going for items to *sell* on *credit bases* (trading), washing of people’s clothes, becoming house girls/house helps to earn some money in order to cater for their needs which includes food [26–30]. Similarly to those studies’ findings, focus group discussions identified selling goods as a way this population creates funds for themselves and their household. Just as the literature identified this population as selling items on a credit basis, the participants in focus groups discussed selling borrowed items, but also their own items when needed.

Family support was a mentioned facilitator for obtaining food in focus group discussions. For participants in focus groups this looked like situation acceptance by family, or boundaries placed by family to provide specific needs of the adolescent. Ultimately, the family support was related to financial support and its influence on food insecurity. However social support specifically is something identified in literature as protective against food insecurity. In a cross-sectional study with pregnant women in Iran, perceived social support, such as informational, emotional, and companionship, was associated with an 81% decrease in odds of household food insecurity [31]. Our findings corroborate previous studies that reported that family support especially from mothers and mother-in-law was key in enabling adolescent pregnant adolescents and mothers acquire food to meet their nutritional need [17, 27, 32].

Transactional sex was mentioned as a way for the adolescents to secure financial support from men in their community. With this phenomenon, men provide goods or money for food to the adolescent girl in exchange for sex. This is usually informal and may happen in the presence or absence of some type of relationship. Additionally, some adolescents mentioned they felt pressured by their families to seek assistance from men to ease the financial burden on the household. This need to find food or money can also be the reason the adolescents are in their current position of pregnancy or motherhood. This trend is supported by literature reporting positive associations between food insecurity and transactional sex among adolescent girls and young women [33]. This could be for a variety of reasons, including a way to gain financial support to cope with food insecurity. Studies in various African countries found that female adolescents were more likely to have ever partook in transactional sex if they experienced food insecurity or had limited assets [33, 34].

### Coping Strategies Employed to Meet Nutritional Needs

Participants mentioned providing services, pawning personal items, trading/selling, borrowing, and meal stretching as strategies they used to get food when access to food and money was limited. The strategy of providing services is similar to a trend seen in the other themes, the ability to work and earn money helps this population provide food for their household. Providing services is another way to accomplish this. In this study, services ranged from hairdressing to seamstress, but they are ways for this population to be self-sufficient. However, it is important to note that most of these jobs do not enable adolescents to be financially secure due to wage instability and inability to negotiate fair pay [35, 36]. Along the same lines, the strategies of pawning personal items, and trading/selling are additional means to obtain money to buy food. In Ethiopia, a study found that 38% of food insecure households reported borrowing money and food [37]. A rural community in Malaysia found that borrowing money to buy food and receiving money from family and friends cushioned against the effects of food insecurity [38]. Similar to findings of this study, other related studies reported that coping strategies employed by adolescents to address food insecurity challenges included selling asset bases, borrowing food and/or money, and purchasing food on credit [29, 39].

Meal stretching was also identified as a coping mechanism of food insecurity among pregnant adolescents and adolescent mothers in Ghana. This strategy has been commonly reported in other studies. Meal stretching was identified as a food insecurity coping strategy in adolescent girls in Indonesia. The girls indicated reducing their portion sizes, eating rice only without any vegetables or side dishes, and skipping meals [40]. A study in south-western Nigeria examining food insecurity coping strategies found that the most common strategies were eating less expensive, less preferred food, and reducing portion sizes [41].

There are a few limitations to this study. First, we observed slightly high dropout rates between enrollment and when the last photovoice session was conducted. However, we found no significant differences in background characteristics between participants who dropped out and those who remained in the study. Nevertheless, the dropout of participants could have resulted in the loss of valuable insights and perspectives that could have been gathered from their photovoice contributions.

A strength of this study includes the utilization of local research assistants that are familiar with the population as focus group facilitators, avoiding any cultural biases. Also, food insecurity questionnaires were administered to the population before the start of the study which showed almost all participants had some experience with food insecurity in the past month. Therefore, participants utilized in the study were the experts in discussing food insecurity experiences in pregnant adolescents and adolescent mothers. Finally, this study provides insight into the experiences and coping strategies of an understudied population.

Pregnant adolescents’ and adolescent mothers’ experience with food insecurity is complex and multifactorial. Based on the findings of this Photovoice study indicating the common barriers, facilitators, and coping mechanisms faced by this population regarding food insecurity, interventions to address food insecurity in pregnant adolescents and adolescent mothers should focus on several scopes. Empowering this population to provide for themselves with trade training and school retention programs to increase employment would be a significant start to help this population, as many of the facilitators to food acquisition are related to the ability to find employment and opportunity, but this population can be limited in this regard without some assistance. Programs aimed at trade training or promoting education in this population should be adapted to be mindful of this population. Mentioned barriers explained that many pregnant adolescents dropped out of school due to feeling unaccepted or judged. To retain this population, program staff should be trained on sensitivity in this subject and an environment should be cultivated in such a way that adolescents can bring their child with them after birth. This type of program could find success being exclusive with pregnant adolescents and adolescent mothers. Also based on barriers mentioned in focus groups, decision makers of the area should consider making efforts to protect the environment and create sustainable gardening initiatives and programs, resulting in an ability for marginalized communities, such as the study population, to improve their situation. This could be initiated on a larger scale to focus on community gardens and protection and restoration of current land, which would also be of benefit to our study population. These efforts and initiatives to address food insecurity in adolescents could have an upstream effect on preventing transactional sex, as a strategy to obtain food, reducing incidence of adolescent pregnancies.
